# Could refeeding-associated gynaecomastia occur during recovery from anorexia nervosa? A hypothesis-generating review

**DOI:** 10.3389/fendo.2026.1910860

**Published:** 2026-08-24

**Authors:** Ali Mohamed Mousa, Mayada Mahmoud Abdalla Mohamed, Mervat Mahmoud-Katheer

**Affiliations:** 1 Family Medicine Department, Kuwait Oil Company, Al Ahmadi, Kuwait; 2 Psychiatry Department, Mansoura University Hospitals, Mansoura, Egypt

**Keywords:** anorexia nervosa, body image, gynaecomastia, hypogonadism, refeeding

## Abstract

International andrology guidance discusses refeeding after starvation as a clinical setting associated with gynaecomastia, based on reports from starvation-refeeding settings outside anorexia nervosa (AN). A targeted search of PubMed, PsycINFO, and Google Scholar, last conducted on 27 July 2026, identified no indexed reports in male patients recovering from AN. We therefore advance the testable hypothesis that transient glandular breast tissue may occasionally occur but remain unrecognised during nutritional recovery; the available evidence does not establish occurrence, prevalence, or clinical significance in AN. Five endocrine transitions provide biological plausibility: non-uniform recovery of the hypothalamic-pituitary-gonadal axis, expansion of the adipose tissue compartment in which aromatase is expressed, a transient leptin rise, possible preservation of adrenal androgen precursor availability, and recovery of insulin-like growth factor 1. Direct human data in male AN support parts of the endocrine trajectory, whereas the proposed link to breast tissue relies largely on indirect human evidence and preclinical studies. Potential under-recognition could arise from misclassification as pseudogynaecomastia, patient-side under-reporting, and follow-up that ends before later endocrine recovery; no clinical detection window has been established. We propose retrospective review, prospective surveillance extending beyond acute refeeding, targeted endocrine evaluation of confirmed glandular tissue, and multicentre studies of prevalence, risk factors, natural history, psychological effects, and treatment engagement.

## Introduction

### Gynaecomastia background

Gynaecomastia is the benign proliferation of glandular tissue in the male breast, typically presenting as a palpable, firm, concentric retro-areolar disc (1, 2). Its pathophysiology reflects a relative predominance of oestrogenic over androgenic action at the breast-tissue level (3). Causes include physiological life stages, hypogonadism, chronic liver disease, hormone-secreting testicular tumours, medications, and idiopathic cases (4, 5). Gynaecomastia must be distinguished from pseudogynaecomastia, in which breast enlargement results from adipose accumulation without glandular proliferation or a palpable retro-areolar disc (6). In adolescent boys and young men, gynaecomastia has been associated with depression, anxiety, lower self-esteem, and psychosocial maladjustment; reported associations with disordered eating are less consistent after accounting for body mass index (7, 8).

### Evidence for refeeding-associated gynaecomastia outside anorexia nervosa

Reports from three historical contexts outside anorexia nervosa (AN) describe gynaecomastia arising after refeeding: Second World War American prisoners of war returning to regular nutrition (9), cirrhotic patients undergoing nutritional rehabilitation (10), and a 1981–1982 outbreak among Haitian entrants to United States detention centres (11). Across these reports, onset occurred during recovery and many cases resolved spontaneously, but study designs, timelines, and endocrine measurements were heterogeneous; they do not establish a single shared mechanism or demonstrate relevance to AN (3, 9–11). The European Academy of Andrology clinical practice guideline includes refeeding after starvation among clinical settings associated with gynaecomastia and discusses hypothalamic-pituitary-gonadal (HPG) recovery after return to a healthy diet (1). In this review, “refeeding-associated gynaecomastia” is used descriptively for glandular breast tissue developing during nutritional recovery; the term does not establish causality.

### Relevance to anorexia nervosa recovery

Although men account for approximately 5-15% of patients with AN, endocrine complications in this population remain incompletely characterised (12). Nutritional recovery from AN shares features with other starvation-refeeding states, but equivalence cannot be assumed. The targeted search described below identified no indexed case report of refeeding-associated gynaecomastia in a male patient with AN. Possible explanations include true absence, extreme rarity, or clinical under-recognition. This review examines under-recognition as one testable possibility rather than presuming it to be the most likely explanation.

### Knowledge gap and review rationale

The relevant guidance remains divided across specialities. Although the European Academy of Andrology guideline discusses refeeding after starvation as a setting associated with gynaecomastia, it does not address recognition during AN recovery (1). Eating-disorder documents from the National Institute for Health and Care Excellence (13), the Academy for Eating Disorders (14), and the Royal College of Psychiatrists (15) address broader endocrine sequelae; the Academy for Eating Disorders guide (14) includes optional luteinising hormone (LH), follicle-stimulating hormone (FSH), oestradiol, and testosterone testing and mentions recovery of gonadal hormone levels, but none specifically addresses glandular breast change during refeeding or provides a dedicated schedule for male sex-steroid monitoring. This hypothesis-generating review therefore maps direct human, indirect human, and preclinical evidence separately; evaluates reasons cases might be missed; and proposes studies capable of establishing occurrence and, separately, clinical importance.

Structured searches of PubMed, PsycINFO, and Google Scholar were last conducted on 27 July 2026 using the Boolean string (“gynaecomastia” OR “gynecomastia” OR “breast enlargement”) AND (“anorexia nervosa” OR “eating disorder*” OR “refeeding” OR “weight restoration”), without language or date restrictions. Reference lists of retrieved articles were screened for additional sources. No indexed reports of refeeding-associated gynaecomastia in male patients with AN were identified by this targeted search. The search was designed to identify reported cases; the mechanistic literature was mapped narratively and should not be interpreted as a systematic review.

## Biological plausibility of refeeding-associated gynaecomastia in male patients with anorexia nervosa: an evidence-graded reconstruction

**Figure 1:**
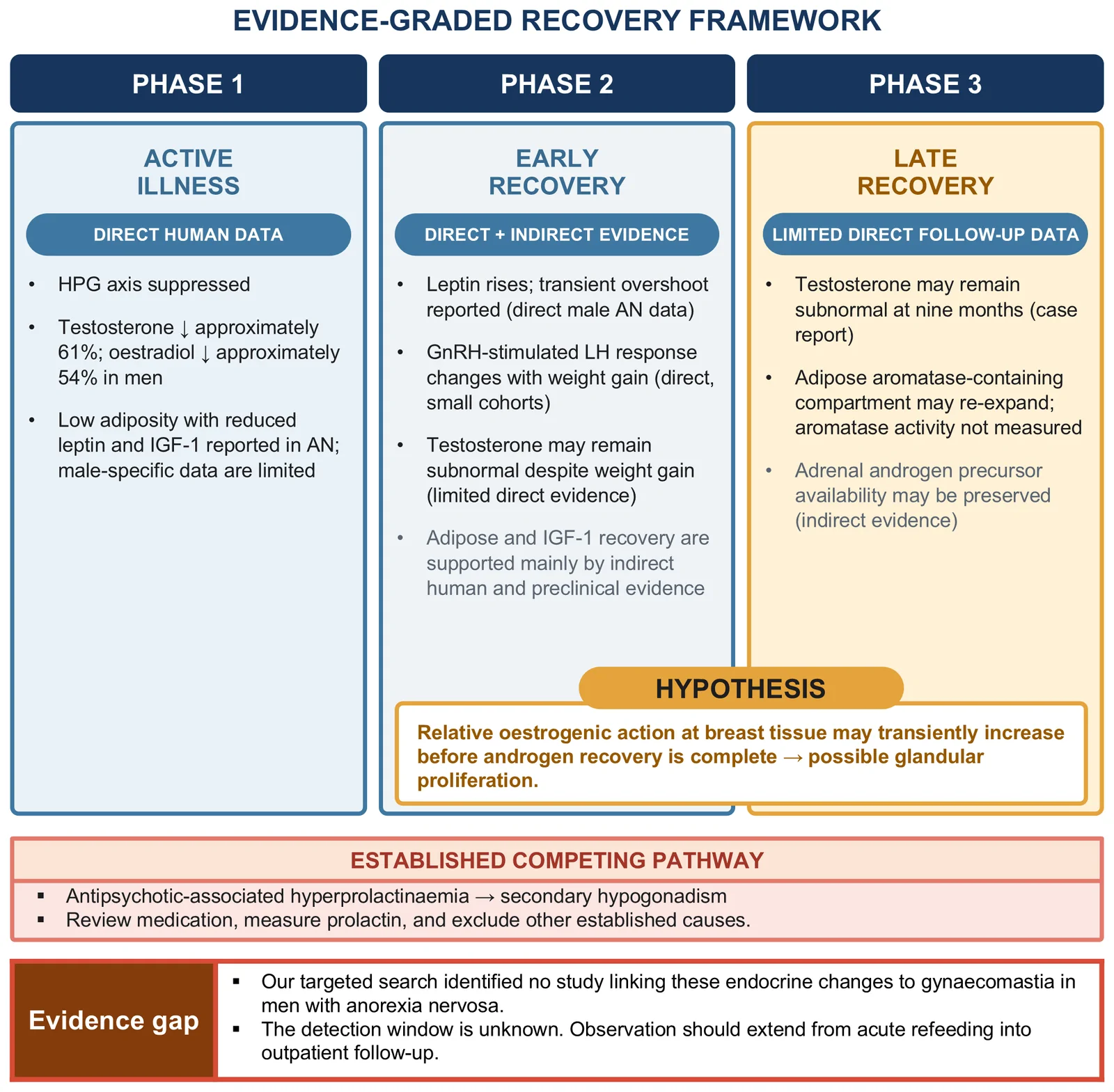
Evidence-graded conceptual framework for possible refeeding-associated gynaecomastia in male patients recovering from anorexia nervosa (AN). Phase 1 summarises endocrine features of active illness (12, 17, 27, 32, 33); Phase 2, endocrine changes during early recovery (20–22, 27); and Phase 3, limited follow-up and indirect evidence relevant to later recovery (3, 16, 24–26, 31). The central band denotes the untested hypothesis, and the lower panel an established competing pathway, antipsychotic-associated hyperprolactinaemia with secondary hypogonadism (1, 62, 63). AN, anorexia nervosa; GnRH, gonadotropin-releasing hormone; HPG, hypothalamic-pituitary-gonadal; IGF-1, insulin-like growth factor 1; LH, luteinising hormone.

Recovery from AN is a dynamic transitional state in which HPG suppression begins to reverse while sex-steroid recovery may lag behind weight gain and gonadotropin normalisation. During the underweight phase, AN can be accompanied by central hypogonadotropic hypogonadism, with low LH and sex-steroid concentrations (12, 16, 17). In a cross-sectional analysis of 31 adult men with AN, Galusca and colleagues (17) reported testosterone and oestradiol concentrations approximately 61% and 54% lower, respectively, than in healthy controls, despite partial preservation of gonadal function. Gonadotropin normalisation may precede sex-steroid recovery. McNab and Hawton (16) described a 27-year-old man whose gonadotropins normalised during weight restoration from 48 kg to 67 kg while serum testosterone remained suppressed at nine-month follow-up. Female AN data provide indirect support for temporal dissociation: Sherman and colleagues (18) reported serum oestradiol below 33 pg/mL in adult women who had returned to more than 90% of ideal body weight, compared with a mid-follicular reference mean of 110 pg/mL; Hussain and colleagues (19) reported persistent oestradiol and testosterone suppression at discharge after partial weight restoration. These observations establish an endocrine context in which relative oestrogenic effects are biologically conceivable, but they do not demonstrate gynaecomastia or its mechanism in male patients with AN. **Figure 1** sets out this endocrine transition by recovery phase, with each phase graded by the evidence available for it.

Five overlapping endocrine transitions are considered below. Their evidentiary status ranges from direct observations of hormone trajectories in male patients with AN to indirect evidence from female AN cohorts, other forms of gynaecomastia, cell studies, and animal models (**Table 1**). Current evidence is insufficient to determine whether any individual pathway, or their combined effects, contributes causally to refeeding-associated gynaecomastia in male patients with AN.

**Table 1:** Evidence grading for endocrine factors proposed to link refeeding with gynaecomastia in male patients with anorexia nervosa.

Endocrine factor	Evidence outside male AN/general physiology	Evidence during recovery from AN	Evidence category and interpretation
Non-uniform HPG-axis recovery	Historical refeeding cohorts document gynaecomastia after starvation/refeeding (9, 11); energy-deficit physiology supports reversible hypogonadotropic hypogonadism (65).	Direct human male AN data: serial studies show changing LH and testosterone with weight restoration, and the GnRH-stimulated LH response changes across low, intermediate, and restored weight states (16, 20–22). Cohorts are small and did not assess breast outcomes.	**Direct human male AN** for non-uniform endocrine recovery; **hypothetical integration** for the LH-aromatisation-gynaecomastia pathway. No causal evidence.
Adipose mass and depot redistribution	Adipose tissue is a site of peripheral aromatisation; restoration expands the available tissue compartment (3, 24). Jacobs reported onset within weeks of improved nutrition, with maximum size reached over the following one to eight weeks (9); Haitian symptoms began around three months after arrival (11).	Indirect human AN data: female cohorts show normalisation of total subcutaneous fat with transiently elevated central adiposity after weight restoration (25, 26, 66). Male and chest-wall-specific data are absent.	**Indirect human** data for adipose expansion; **hypothetical integration** for extrapolation to male chest-wall aromatase activity. The magnitude and timing are unknown.
Leptin rise	*In vitro* MCF-7 breast cancer cell data show leptin-induced aromatase expression and activity, and a small pubertal case-control study reported higher leptin without significant differences in measured sex hormones or the oestradiol-to-testosterone ratio (29, 30).	Direct human male AN data show a steep leptin rise and occasional overshoot during refeeding (27); a single metreleptin case showed reversible testosterone normalisation (28). No breast outcome was reported.	**Direct human male AN** for the leptin trajectory; **preclinical** for breast effects; **hypothetical integration** for the causal link.
Adrenal androgen substrate	Androstenedione is an aromatizable precursor for peripheral oestrogen synthesis; dehydroepiandrosterone contributes through conversion to androstenedione (24).	Cross-sectional AN literature suggests relative preservation of adrenal androgen precursors, but direct longitudinal recovery data in male patients are sparse (12, 17, 31).	**Indirect human** data are limited for adrenal precursor preservation; **hypothetical integration** for the substrate effect. Substrate availability has not been linked to breast tissue change.
IGF-1 recovery	IGF-1 rises with caloric rehabilitation; preclinical mammary studies show a role in ductal morphogenesis (32–34).	Human AN studies support IGF-1 recovery during nutritional rehabilitation, but male-specific trajectories and breast outcomes are limited (32, 33).	**Indirect human** evidence for IGF-1 recovery, largely from female cohorts; preclinical evidence for breast effects; **hypothetical integration** for the causal contribution.
Antipsychotic-associated hyperprolactinaemia with secondary hypogonadism	Not proposed as the mechanism in historical starvation-refeeding cohorts.	Atypical antipsychotics are used in some inpatient AN settings (62), and antipsychotic-induced hyperprolactinaemia can cause secondary hypogonadism and thereby contribute to gynaecomastia through reduced androgen action (1, 63).	**Established competing diagnosis**. Review medications, measure prolactin, and assess alternative causes in every confirmed case.
Synthesis and case-finding framework	Historical reports show variable onset after refeeding and do not define a universal endocrine timeline (9–11).	Limited male AN data suggest that sex-steroid recovery can lag behind weight restoration (16, 20, 21), but our targeted search identified no indexed cases or onset data.	**Hypothetical integration**: case-finding framework. No detection window is established; observation should span acute refeeding and later follow-up without presupposing onset timing.

Evidence category key (greyscale-safe through wording): Direct human male AN; Indirect human; Preclinical; Hypothetical integration; Established competing diagnosis. Bold text in the final column names every category that applies to that row. Cell shading follows the strongest evidence class named in the row: green where the endocrine change is directly evidenced in male AN, cream where it rests on indirect or preclinical evidence, salmon for the established competing diagnosis, and mauve for the concluding synthesis row.

AN, anorexia nervosa; GnRH, gonadotropin-releasing hormone; HPG, hypothalamic-pituitary-gonadal; IGF-1, insulin-like growth factor 1; LH, luteinising hormone.

First, components of the HPG axis may recover non-uniformly during weight restoration. The principal direct human evidence comes from two overlapping reports, by Wheeler and colleagues (20) and Crisp and colleagues (21), describing the same twelve men with AN admitted to a single London unit between 1974 and 1979, in which individual hormones were measured in subsets of the cohort only. Basal LH and testosterone were significantly correlated with body weight and rose during refeeding, whereas FSH did not correlate with weight and remained low or low-normal. Across repeated gonadotropin-releasing hormone (GnRH) stimulation testing in five men, the LH response was subnormal below 40 kg, normal between 40 and 45 kg, exaggerated between 45 and 50 kg, and normal above 50 kg, whereas the FSH response remained within the normal range on all but one occasion (20). The youngest patient showed an exaggerated LH response at 46 kg despite being at 101% of matched population mean weight, supporting the investigators’ conclusion that absolute body weight tracked this transition more closely than percentage matched population mean weight. Circulating testosterone during this interval was characterised as 4–6 nmol/L, below the adult reference range, although the two reports interpret its role differently: Wheeler and colleagues proposed an enlarged pituitary store of LH with transiently unbalanced homeostatic control, whereas Crisp and colleagues (21) attributed the exaggerated response to reduced negative feedback from low testosterone. In a separate cohort, Lemaire and colleagues (22) reported diminished testosterone at maximum weight loss followed by recovery-phase increases in testosterone, oestradiol, gonadotropins, and GnRH responsiveness; however, only testosterone correlated significantly with corpulence index, whereas oestradiol, LH, and FSH did not. Collectively, these small studies are consistent with non-uniform HPG-axis recovery. The further hypothesis that transient recovery of LH secretion or responsiveness could enhance gonadal aromatase activity before circulating testosterone fully normalises is biologically plausible (1, 23) but remains untested in male patients with AN.

Second, adipose tissue expands during nutritional rehabilitation, potentially increasing the tissue compartment available for peripheral aromatisation relative to the severely depleted state (3, 24). The AN evidence is indirect for male patients: cross-sectional whole-body imaging in short-term weight-restored adult women shows total subcutaneous adipose tissue normalising to control levels while visceral adipose tissue and the trunk-to-extremity fat ratio remain elevated (25). Longitudinal magnetic resonance imaging suggests that this central-adiposity phenotype resolves over approximately one year of sustained weight maintenance (26). Whether these patterns occur in male patients, alter aromatase activity, or apply specifically to chest-wall adipose tissue is unknown. Expansion of an aromatase-containing tissue compartment before full testicular recovery is therefore plausible, but aromatase activity has not been measured in this setting.

Third, leptin may act as a permissive signal. Direct male AN data show that leptin rises steeply and can reach or surpass age- and sex-specific upper reference percentiles during refeeding, alongside changes in gonadotropins, testosterone, and the free androgen index (27). In a single male adolescent with severe AN, off-label metreleptin treatment was accompanied by rapid testosterone normalisation during dosing, followed by a fall toward baseline when the dose was reduced (28). Evidence connecting leptin to breast tissue is indirect: *in vitro* work in MCF-7 human breast cancer cells found that leptin increased aromatase mRNA, protein, and enzymatic activity (29), which does not establish an effect in normal male breast tissue. Dundar and colleagues (30) found higher leptin in 20 non-obese boys with pubertal gynaecomastia than in 20 age-, pubertal-stage-, and body-mass-index-matched controls, whereas the measured sex hormones and oestradiol-to-testosterone ratio did not differ significantly. These findings support leptin as a candidate permissive signal, not a proven cause of refeeding-associated gynaecomastia.

Fourth, adrenal androgen precursors could provide aromatisable substrate. Androstenedione is a major peripheral precursor for oestrone in men, and dehydroepiandrosterone contributes through conversion to androstenedione (24). In women with AN, dehydroepiandrosterone sulphate may be relatively preserved compared with gonadal androgens, but the pattern is affected by oral contraceptive exposure and has not been characterised longitudinally in male refeeding cohorts (31). Galusca and colleagues (17) reported partial preservation of inhibin B in men with AN, but this does not establish preservation of adrenal precursor supply. The proposed substrate effect is therefore indirect.

Fifth, insulin-like growth factor 1 (IGF-1), which is suppressed during active AN, rises with nutritional rehabilitation (32, 33). Human AN studies, largely in females, support this recovery trajectory. Female mouse studies show that IGF-1 is required for normal pubertal mammary terminal end-bud formation and ductal morphogenesis (34), but no human study links recovery of IGF-1 to male breast proliferation after AN. IGF-1 is therefore a biologically plausible permissive factor rather than a demonstrated mechanism.

Sex hormone-binding globulin (SHBG) warrants distinct consideration because nutritional state can alter SHBG concentrations and thereby influence circulating sex-steroid availability (35). Although SHBG measurement is important for interpreting total testosterone and estimating free or bioavailable testosterone, direct evidence in male AN is limited. Wheeler and colleagues (20) measured SHBG in five male patients, found no correlation with body weight, and reported normal concentrations in four and a slight elevation in one. SHBG did not decline significantly despite rising testosterone, whereas SHBG normally decreases with advancing puberty in boys (36, 37). Wheeler and colleagues (20) proposed three possible explanations: the relatively short duration of testosterone recovery compared with puberty, testosterone concentrations remaining above prepubertal levels, or a compensatory change in plasma oestrogen, which was not measured. Because SHBG binds testosterone more avidly than oestradiol (5), persistently unchanged SHBG could, in principle, limit the rise in free or bioavailable testosterone relative to oestradiol; however, neither oestradiol nor free hormone concentrations were measured, and this possibility remains untested during refeeding in male patients with AN. Female AN studies report elevated SHBG in some cohorts, with a decline during nutritional rehabilitation (38, 39), but extrapolation to male refeeding is uncertain, and the available evidence does not establish SHBG as an independent driver.

## Potential clinical implications

Gynaecomastia can carry substantial psychological significance because breast development may conflict with an individual’s body-image goals. In a propensity-matched retrospective cohort of adolescents with persistent gynaecomastia and no previous psychiatric diagnosis, those who underwent mastectomy had lower incidences of subsequent psychiatric morbidity, anxiety, and suicidal ideation or self-harm than those managed non-operatively; however, the observational design does not establish that surgery caused these differences (40). Fisher and Fornari (41) described two adolescent boys in whom distress about gynaecomastia was temporally associated with disordered eating: one restricted intake and induced vomiting in an attempt to reduce breast size, whereas the other used self-induced vomiting because his breasts were not “going away” despite vigorous exercise. These case reports do not establish a physiological pathway from refeeding to gynaecomastia in male patients with AN and do not quantify relapse risk; rather, they illustrate that unexpected breast development can become highly salient in some young people.

If refeeding-associated gynaecomastia occurs, it could therefore influence treatment engagement in a subset of male patients recovering from AN, particularly when the illness is organised around muscularity, fear of weight gain, or concerns about masculinity (42, 43). Whether it would impair treatment engagement, increase relapse risk, or have no measurable effect is unknown and requires direct study rather than extrapolation from adolescent gynaecomastia cohorts or isolated case reports.

The psychological meaning of breast tissue change may vary substantially between individuals. It may be particularly salient for patients with pre-existing body-image concerns, chest dysphoria, or eating-disorder behaviours focused on chest shape. The same physical change may also carry different meanings across gender identities and treatment goals; this is a person-centred clinical consideration rather than evidence for a distinct endocrine mechanism. Clinicians should use patient-preferred terminology, explore individual concerns and goals without presuming distress, and coordinate with clinicians experienced in gender-affirming care when appropriate (44, 45).

Clinical awareness may nevertheless be useful if it is framed without implying that gynaecomastia is an expected consequence of refeeding. Psychoeducation can encourage patients to report breast tenderness or tissue change while explaining that several causes are possible. When palpable glandular tissue is confirmed, evaluation and management should follow established gynaecomastia guidance, including assessment for alternative causes and attention to the individual’s psychological and gender-related needs (1, 5). Although spontaneous resolution is common in several forms of gynaecomastia, the natural history of any refeeding-associated cases in AN remains unknown.

## Potential reasons for under-recognition

If refeeding-associated gynaecomastia occurs during recovery from AN in male patients, three factors could contribute to under-recognition.

First, visual misclassification as pseudogynaecomastia. Adipose tissue increases during nutritional rehabilitation, and female AN cohorts show normalisation of total subcutaneous adipose tissue with weight restoration (25). Visual inspection alone cannot reliably distinguish adipose accumulation from a palpable retro-areolar glandular disc (5). Without a consent-based focused examination, glandular tissue could be attributed to ordinary weight gain or left undocumented when symptoms or visible change arise.

Second, patient-side under-reporting. Some male patients with AN may interpret chest changes as confirmation of feared weight gain rather than as a discrete symptom, particularly where muscularity and masculinity concerns are prominent (42, 43). Shame, treatment disengagement, or the patient’s own attribution of the change to adiposity could reduce spontaneous reporting. This explanation is plausible but has not been tested.

Third, insufficient follow-up duration to capture later endocrine events. Jacobs reported cases beginning within weeks after improved nutrition, with individual swellings reaching maximum size over the following one to eight weeks (9). In the Haitian cohort, symptomatic cases generally began around three months after arrival in the United States (11). Limited male AN data show that testosterone recovery can lag behind weight gain and gonadotropin normalisation (16, 20–22). These disparate observations do not justify a fixed detection window. They support only the hypothesis that case finding should include acute refeeding and subsequent outpatient follow-up, with onset timing treated as unknown until observed cases are available.

## Limitations and knowledge gaps

The hypothesis is not supported by documented cases in male AN and cannot establish existence, frequency, causality, or clinical burden. Four uncertainties require explicit acknowledgement and define the evidence needed to advance or reject the model.

First, Brody and Loriaux (46) proposed exposure to the pyrethroid d-phenothrin as an alternative aetiology for the Haitian outbreak that partially underpins the refeeding hypothesis. Yamada and colleagues (47) did not reproduce oestrogenic, androgenic, or anti-androgenic activity in rat uterotrophic and Hershberger assays at the Organisation for Economic Co-operation and Development limit dose of 1000 mg/kg/day. These animal data weaken, but do not by themselves exclude, the human exposure hypothesis.

Second, most proposed mechanistic components rely on indirect evidence from female patients with AN, pubertal gynaecomastia, cellular studies, or murine mammary development rather than direct observations in male patients with AN. The absence of any correlation between SHBG and body weight in five male patients reported by Wheeler and colleagues (20) weakens extrapolation from female trajectories. A further male-specific consideration is compulsive exercise, which is prominent in some male presentations (42, 43). Whether it modifies truncal fat restoration or aromatase-relevant tissue recovery is unknown.

Third, the evidence linking gynaecomastia to disordered eating is limited and sensitive to adjustment for adiposity. Nuzzi and colleagues (8) found that higher Eating Attitudes Test-26 scores in adolescent boys with gynaecomastia were no longer significant after adjustment for body mass index, suggesting that the disordered-eating signal in that cohort may have reflected adiposity. In contrast, lower self-esteem and several psychosocial quality-of-life domains remained significantly worse independent of body mass index.

Fourth, cortisol is a candidate modifier whose direction during male AN recovery is uncertain. Glucocorticoid signalling can induce aromatase activity in female-derived adipose stromal cells and human breast stromal cells under specific experimental conditions (48–50), but this does not establish an *in vivo* effect in men. Chronic hypercortisolism in Cushing syndrome suppresses the gonadal axis in a high proportion of men (51). Hypogonadotropic hypogonadism was present in 75% at diagnosis in one series (52), and hypogonadism, defined by a total testosterone below 3.0 ng/mL, in 83%, 61%, and 33% of men with ectopic adrenocorticotropic hormone secretion, Cushing disease, and adrenal Cushing syndrome, respectively, in another (53). Major Cushing syndrome reviews and guidelines do not identify gynaecomastia as a characteristic clinical feature (54, 55), and current gynaecomastia guidance, reviews of drug-induced gynaecomastia, and pharmacovigilance analyses do not establish glucocorticoid excess as a common cause (5, 56–60). In male abdominal subcutaneous preadipocytes, cortisol has also been reported to suppress rather than induce aromatase activity, with responses varying by depot, sex, cell stage, and co-exposure to insulin (61). The net effect in male breast tissue during refeeding therefore requires direct measurement and should not be inferred from isolated *in vitro* findings.

Prospective null findings would be informative only if assessment is systematic, follow-up is sufficiently long, and the cohort is large enough to detect an uncommon event. Failure to identify cases in a small or symptom-triggered sample would narrow but not definitively refute the hypothesis.

## Practical steps to evaluate the hypothesis

First, existing refeeding cohorts of male patients with AN should undergo retrospective review for documented breast enlargement, mastalgia, chest-wall concerns, focused examination, imaging, or endocrine evaluation. Where breast ultrasonography was performed, the reported pattern may indicate the approximate duration of glandular change at the time of imaging, since nodular appearances have been associated with symptoms of less than one year and dendritic appearances with longer-standing disease (5). Historical cohorts described by Lemaire and colleagues (22) (n = 8), Wheeler and colleagues (20) (n = 12; hormone data in 10), and Wabitsch and colleagues (27) (n = 3) are starting points, but the absence of documentation cannot be equated with a negative finding.

Second, prospective studies should record baseline chest findings and use sensitive, consent-based inquiry about tenderness and breast change at predefined points during acute refeeding and later follow-up. When symptoms or visible change are present, trained palpation should assess for a concentric retro-areolar glandular disc (5); targeted ultrasonography can help in equivocal cases (1). Standardised assessment is needed because visual inspection alone cannot distinguish glandular tissue from adipose accumulation.

Third, when palpable glandular tissue is confirmed, protocols should record LH, FSH, morning total testosterone, SHBG and albumin for calculation of free testosterone, oestradiol, prolactin, β-human chorionic gonadotropin, and relevant alternative-cause investigations, together with clinical and ultrasound evaluation of the testes. Androstenedione, listed in current guidance as a second-line test, is also relevant as the aromatisable precursor underlying the proposed adrenal contribution (5). The oestrogen-to-androgen balance is more informative than an isolated testosterone value (1). Prolactin should be interpreted alongside a medication review for antipsychotic-associated hyperprolactinaemia and secondary hypogonadism (1, 62, 63). Where available, liquid chromatography-tandem mass spectrometry is preferred for oestradiol in the male range (64). Cortisol may be included as an exploratory measure because its role is uncertain, as discussed above.

Fourth, multicentre denominator-based studies should distinguish occurrence from clinical significance. Outcomes should include incidence or prevalence, baseline and treatment-related risk factors, time to onset and resolution, laterality, pain, spontaneous regression, need for treatment, psychological distress, eating-disorder symptoms, treatment adherence, and relapse. Qualitative work should include participants with diverse gender identities and chest-related goals. Identification of cases alone would not justify routine screening or intervention without evidence of burden and benefit.

## Conclusion

Reports from non-AN starvation-refeeding contexts and endocrine observations in AN make refeeding-associated gynaecomastia biologically plausible, but they do not establish that it occurs in male patients with AN. Our targeted search identified no indexed cases, but this search-bound absence is not proof of non-occurrence. The central contribution of this review is a falsifiable case-finding framework, not a claim that the condition is expected or that it follows an established timeline. Retrospective review and prospective, consent-based assessment across acute and later follow-up can determine occurrence; only larger studies can establish prevalence, risk factors, natural history, and clinical significance. If adequately designed cohorts do not identify cases, that null result will constrain the hypothesis and may reveal a meaningful divergence from other starvation-refeeding states.

## Data Availability

The original contributions presented in the study are included in the article/supplementary material. Further inquiries can be directed to the corresponding author.
